# Association between thymoma and persistent hypothermia: a case report

**DOI:** 10.1186/1752-1947-3-73

**Published:** 2009-10-12

**Authors:** Robin H Johns, Alistair K Reinhardt

**Affiliations:** 1 Chest Clinic, Whipps Cross University Hospital NHS Trust, London E11 1NR, UK

## Abstract

**Introduction:**

Thymomas are rare, slow-growing tumours that present in a variety of ways such as incidental findings on chest radiographs following symptoms of cough and dyspnoea. Thymomas may also present with symptoms due to intrathoracic spread such as superior vena cava obstruction, or with symptoms of an associated paraneoplastic disorder. Such paraneoplastic disorders are typified by the generation of autoantibodies directed against a variety of self antigens including myasthenia gravis, neuromyotonia, and hypogammaglobulinaemia.

Significant hypothermia in association with thymoma has been described previously in one published case report. The basis for hypothermia in that case was not clear, but was postulated to relate to abnormal central thermal regulation and was resolved completely following treatment with intravenous gammablobulin, thus suggesting an autoimmune aetiology.

**Case presentation:**

We present the case of an 88-year-old man with Type A thymoma and persistent hypothermia. An extensive investigation of the hypothermia revealed no aetiology other than the thymoma itself. Symptoms of hypothermia were treated effectively with passive and active external rewarming. The patient's dyspnoea was much improved by intercostal drainage of a left-sided pleural effusion and talc pleurodesis. He was not offered definitive treatment for the thymoma in view of its relatively favourable prognosis, and because his symptoms were well controlled at the time of discharge.

**Conclusion:**

We suggest that the possibility of thymoma be investigated once the more common causes of hypothermia have been excluded in an appropriate clinical context. To the best of our knowledge, this is only the second published case report describing such an association.

## Introduction

The thymus is a small anterosuperior mediastinal organ involved in the processing and maturation of T lymphocytes. The thymus gland grows from birth until puberty, when it reaches a maximum weight of approximately 40 grams. It subsequently atrophies but persists in an atrophic state into old age.

Thymomas are the most common neoplasm to affect the thymus gland, with an incidence of 0.15 per 100,000. The probability of developing the condition increases as one reaches the eighth decade of life and is more pronounced in men [1]. Thymomas often present as incidental findings on chest radiographs performed in asymptomatic patients. Whenever present, symptoms typically include cough and dyspnoea.

Thymomas may also present with symptoms secondary to intrathoracic spread such as superior vena cava obstruction or with symptoms of an associated paraneoplastic disorder such as myasthenia gravis. Over 90% of thymomas occur in the thymic tissue in the anterosuperior mediastinum. However, thymomas have also been reported to develop in the neck, trachea, thyroid, parathyroid, pericardium, heart, pleura and lung, without evidence of thymic lesion in the anterosuperior mediastinum [2].

Thymomas are typically slow-growing tumours and, relative to neoplasms of the lung, bowel and pancreas, have a considerably better five-year survival rate. The World Health Organization (WHO) pathological classification recognizes five histological types of thymoma: Types A, AB, B1, B2 and B3. This classification recognizes morphological similarities between normal thymic epithelial cells and neoplastic cells. Its prognostic significance [3] is detailed in Table 1. Prognosis also relates to tumour stage, and the most widely utilized staging, also outlined in Table 1, is that defined by Masaoka [4].

**Table 1:** Pathological classification and clinical staging of thymoma

WHO Thymoma classification	5-year survival rate (%)
Type A	100

Type AB	93

Type B1	89

Type B2	82

Type B3	71

	

**Masaoka Clinical Stage**	**Macroscopic or Microscopic Features**

I	Encapsulated tumour. No evidence of capsule invasion

IIa	Macroscopic invasion into surrounding fatty tissue or mediastinal pleura

IIb	Microscopic invasion into the capsule

III	Macroscopic invasion into neighbouring organs including pericardium, major vessels or lung.

IVa	Pleural or pericardial dissemination.

IVb	Lymphogenous or haematogenous metastasis.

The optimum treatment for thymoma is surgical excision, particularly for small, encapsulated tumours. If the tumour has invaded locally, it may be technically possible to resect pleural or pericardial structures. However, the mortality risk associated with potentially major surgery may be greater than that which is attributable to the tumour itself. Adjuvant radiotherapy generally follows when surgery is undertaken for stage III and IV thymomas. For unresectable disease or in those patients not considered sufficiently fit for surgery, combination chemotherapy with platinum-based regimes may be used.

Paraneoplastic disorders associated with thymoma are mediated by the generation of autoantibodies directed against a variety of self antigens. Myasthaenia gravis occurs in up to 50% of patients with thymoma. Autoantibody-mediated destruction of nicotinic acetylcholine receptors at the neuromuscular junction causes sporadic skeletal muscle weakness. Some patients with thymoma-associated myasthenia gravis also develop an inflammatory myositis of the skeletal and cardiac muscles. In neuromyotonia, autoantibodies are directed against voltage-gated potassium channels in peripheral nerves. The symptoms of neuromyotonia are myokymia, muscle stiffness, cramps and, occasionally, muscle hypertrophy.

Red cell aplasia occurs in 5% of patients with thymoma [5] and is associated with autoimmune-mediated suppression of erythropoiesis in the bone marrow. Hypogammaglobulinaemia (Good's syndrome) also occurs in association with thymoma at an incidence of 6% to 11% [6]. In such cases, hypogammaglobulinaemia is accompanied by deficiencies in B cells and CD4 cells, and is typically associated with recurrent sinopulmonary infection. Pemphigus is another association of thymoma, wherein autoantibodies are directed against the epidermal proteins of the skin.

Hypothermia is a reduction in core body temperature to below 35°C, which can be classified as mild (32°C to 35°C), moderate (28°C to 32°C), or severe (<28°C). Low core body temperatures are best measured with a low-reading thermometer. Hypothermia typically occurs following an exposure to cold ambient temperatures when the body heat is lost through radiation, conduction, convection, evaporation and respiration. However, there are a variety of other causes of hypothermia. These can be categorized according to the mechanism shown in Table 2. It is particularly important to exclude sepsis as a cause of hypothermia in the elderly, who register an incidence that is particularly high.

**Table 2:** Causes of hypothermia (from [10])

Likely Mechanism	Cause of Hypothermia
**Decreased heat production**	Hypothyroidism

	Hypopituitarism

	Hypoadrenalism

	Malnutrition

	Hypoglycemia

	Neuromuscular inefficiency

	

**Increased heat loss**	Accidental (exposure to cold ambient temperature)

	Drug-induced vasodilatation

	Burns

	Exfoliative dermatitis

	Severe psoriasis

	Iatrogenic (e.g. treatment of pyrexia, large volume infusions of non-warmed fluid)

	

**Impaired thermoregulation**	Central nervous system trauma

	Acute spinal cord transection

	Stroke/Intracranial haemorrhage

	Central nervous system tumour

	Parkinson's disease

	Multiple sclerosis

	Wernicke's disease

	

**Combination of mechanisms**	Sepsis

	Ethanol intoxication

	Hypnotics

	Phenothiazines

When core body temperature begins to fall, homeostatic mechanisms act to prevent heat loss and to increase heat production. Heat loss is prevented by peripheral vasoconstriction and behavioural responses such as applying additional layers of clothing. Heat production is achieved by shivering accompanied by an increased production of thyroxine and adrenaline. These thermoregulatory mechanisms are deficient in the elderly. Consequently, they are more susceptible to hypothermia.

The typical initial clinical manifestations of hypothermia are lethargy, confusion, tachycardia, tachypnoea and shivering. In more severe hypothermia, impaired judgement, ataxia, diminished reflexes, hypotension, bradycardia, decreased respiratory rate, delirium and coma may occur. Biochemical and haematological investigation may reveal evidence of hypo- or hyperkalaemia, renal impairment and coagulopathy. Echocardiogram abnormalities include prolongation of PR, QT, and QRS intervals, J-point elevation, atrial fibrillation, and 2^nd ^or 3^rd ^degree heart block. Ventricular fibrillation and asystole occur as preterminal events.

The treatment of hypothermia requires rewarming. Passive external rewarming, which depends on heat generation by shivering and an increased metabolic rate, involves insulating the patient with covers to prevent heat loss. Meanwhile, active external rewarming employs externally applied heating devices such as heating pads and hot air blankets. Lastly, active internal rewarming is used in cases of severe hypothermia, and includes techniques such as ventilation with warmed humidified air and the infusion of warmed fluids. Alternatively, extracorporeal techniques such as cardiopulmonary bypass may be employed.

## Case presentation

An 88-year-old Caucasian man with a two-month history of breathlessness was referred to our hospital. His chest radiograph showed a large, left-sided pleural fluid collection. He was admitted for further investigation.

The patient was hypertensive and had undergone transurethral resection of a grade 1 adenocarcinoma of the prostate 23 years prior to presentation. His serum prostate-specific antigen (PSA) level had not risen subsequently. His medication included amlodipine and bendroflumethiazide. He smoked 1 to 2 cigarettes per day until 5 years previously, and also drank alcohol infrequently. He mobilized with a walking aid and was able to ascend a single flight of stairs. He was a retired clerical worker.

Physical examination revealed a body temperature of 35.1°C, measured using a tympanic thermometer and checked with a low-reading thermometer. He had no fingernail clubbing, palpable lymphadenopathy or manifestation of cardiac failure. His abdominal examination results were unremarkable. There was neither papilloedema nor lateralizing signs on neurological examination to suggest the possibility of a structural central nervous system lesion or stroke. A computed tomography (CT) brain scan showed that he had a small lacunar infarct in his left periventricular region with intracranial appearances within normal limits. His lower limbs demonstrated chronic changes of venous congestion. No evidence of any skin wound or cellulitis was found.

He had a good appetite prior to hospital admission and there was no clinical or biochemical evidence of malnutrition (serum calcium, phosphate, vitamin B_12 _and folate levels were all normal). Other blood tests revealed normal haemoglobin (14.1 g/dL) and white cell count (8.5 × 10^9^/L), with a mild elevation of creatinine and a C-reactive protein (CRP) level of 53.1. Following the identification of pseudomonas based on swabs taken from his lower limbs, he was treated with ciprofloxacin. Blood cultures were negative for bacterial growth and his CRP was noted to have normalized.

An intercostal tube was inserted to drain pleural fluid, and pleural biopsies were then performed. The fluid was a blood-stained exudate and contained no organisms, leucocytes or malignant cells. Pleural biopsies contained mesothelial cells and haemosiderin-laden macrophages but did not show any features of malignancy. Acid and alcohol fast bacilli were not cultured from any pleural specimens.

**Figure 1:**
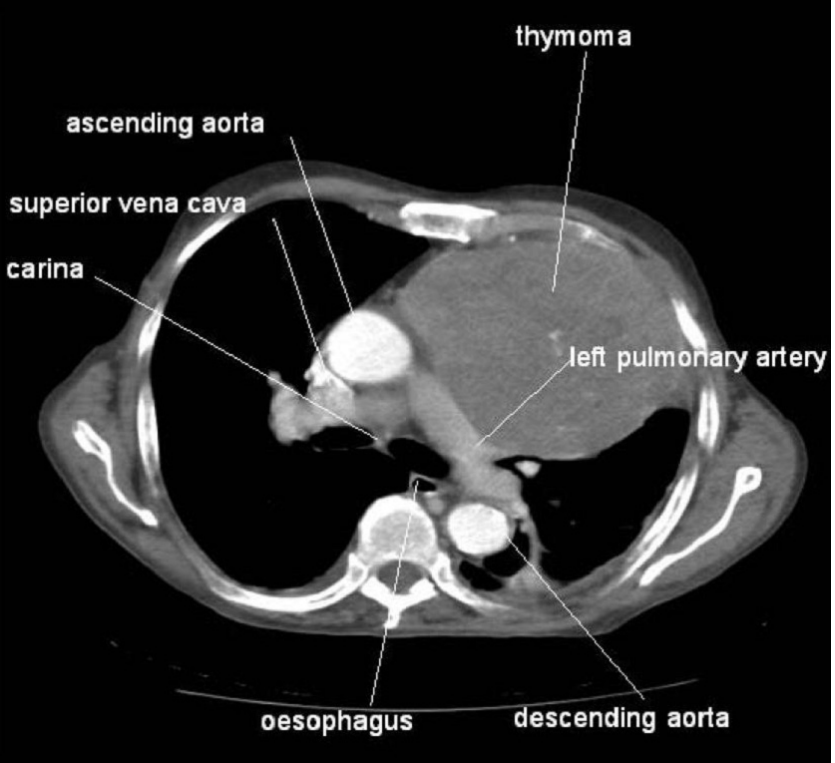
**Computed tomography image of thymoma**. Figure shows image at level of carina, with large left-sided thymoma. Major mediastinal structures are labelled for orientation purposes.

A CT scan demonstrated a 12.5 × 10 cm mass within the patient's left hemithorax contiguous with the anterior and lateral mediastinum, a small pericardial effusion, and a residual left pleural effusion with associated atelectasis. An ultrasound guided core biopsy of the mass was performed. A CT image of the thymoma at the level of the carina is shown in Figure 1. Histological and immunohistochemical examination demonstrated Type A thymoma (Table 1). Radiological findings suggest that this was a Stage IVa tumour according to the Masaoka staging system. A talc pleurodesis was performed before the intercostal drain was removed, and the pleural fluid did not reaccumulate. The dyspnoea resolved. We did not offer the patient any further treatment in view of the relatively good prognosis and his lack of remaining symptoms. There was no evidence of myasthenia gravis, neuromyotonia, or pemphigus. The patient's immunoglobulin levels were normal and his acetylcholine receptor antibodies were negative.

**Figure 2:**
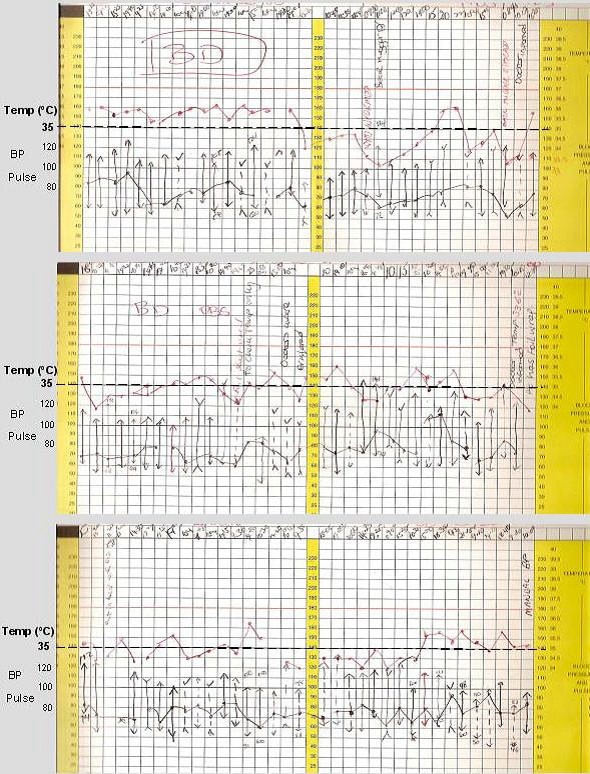
**Hypothermia recorded on observation charts**. This image shows the representative observation charts (of 6) showing persistent hypothermia with temperatures recurrently dropping below 35°C.

However, the patient remained intermittently hypothermic for another four weeks and active external rewarming was required on some occasions. Despite this, his temperature fell to as low as 32.8°C. The ambient ward temperature was at least 22°C at all times. Representative temperature recordings from his observation charts are shown in Figure 2. His amlodipine medication was stopped in case its peripheral vasodilatory effect was contributing to hypothermic condition. Results of his thyroid function and hypothalamic-pituitary-adrenal axis tests were normal. There was no clinical evidence of sepsis throughout this period. His white cell count and CRP remained within normal limits and no evidence of bacteremia was found following repeat blood cultures. His condition subsequently remained stable and his symptoms were well controlled at the time of his discharge from our hospital.

## Discussion

Our patient had no evidence of any common precipitant of hypothermia (Table 2) such as endocrine dysfunction, malnutrition, neurological disease, or sepsis. Pseudomonas was isolated from skin swabs, but no wound infection or ulcer was apparent. Pseudomonas is not a common commensal of normal skin, although it does colonize moist skin and chronic ulcers. It is possible that pseudomonal colonization may have resulted from previous healed venous ulcers.

Thymoma was associated with hypothermia in a previous case report [7]. In this report, the resolution of hypothermia following treatment with intravenous gammablobulin treatment (0.4 g/kg per day for five days) suggested an immunological basis such as circulating autoantibodies. Interestingly, hypothermia has also been described in limbic encephalitis associated with autoantibodies [8]. In some patients with this condition, the presence of voltage-gated potassium channel antibodies is also associated with other symptoms of autonomic dysfunction including somnolence, sweating, hypersalivation and appetite alteration. These symptoms improve following a reduction of autoantibody titres induced by immunotherapy.

Inflammatory changes within the hypothalamus have also been observed on magnetic resonance imaging brain scans. Hence, it is plausible that in such patients, hypothermia results from autoantibody induced hypothalamic injury and altered thermoregulation.

It is not possible to confidently conclude the basis for hypothermia in our patient since we did not find any evidence of a common precipitant. It is tempting to speculate that an autoantibody associated paraneoplastic syndrome might be the cause of hypothermia in our patient, although we did not note the presence of any other paraneoplastic features. Our patient's immunoglobulin levels were normal and his acetylcholine receptor antibodies were negative. Our patient did not receive immunoglobulin, plasmapheresis or immunosuppression, and the thymoma was not excised. Therefore, his hypothermia could not be expected to resolve if its basis was a paraneoplastic autoimmune process, unless this regressed spontaneously. However, such spontaneous resolution can occur, and has been described for both thymoma [9], and associated autoantibody mediated conditions such as myasthenia gravis [10].

## Conclusion

In conclusion, we propose that hypothermia might occur as a paraneoplastic phenomenon in the context of thymoma. We would obviously advocate careful and thorough exclusion of the more common causes of hypothermia, such as sepsis, prior to reaching this conclusion, as failure to treat these could have dire consequences.

## Abbreviations

CRP: C-reactive protein; CT: computed tomography; PSA: prostate-specific antigen; WHO: World Health Organisation.

## Competing interests

The authors declare that they have no competing interests.

## Consent

Written informed consent was obtained from the patient's next of kin for publication of this case report and any accompanying images. A copy of the written consent is available for review by the Editor-in-Chief of this journal.

## Authors' contributions

RHJ was involved in case review, literature review and in drafting the manuscript. AKR was involved in manuscript critique and manuscript editing. All authors read and approved the final manuscript.
